# Unveiling Metabolic Capability and Growth Adaptation of *Monascus purpureus* NP1 Through Genomic Sequencing and Comparative Analysis

**DOI:** 10.3390/ijms27083670

**Published:** 2026-04-20

**Authors:** Haisu Hu, Preecha Patumcharoenpol, Kangsadan Boonprab, Amornthep Kingkaw, Yu Zhang, Kamonporn Masawang, Wanwipa Vongsangnak

**Affiliations:** 1 Interdisciplinary Graduate Programs in Bioscience, Faculty of Science, Kasetsart University, Bangkok 10900, Thailand; haisu.h@ku.th; 2 Department of Zoology, Faculty of Science, Kasetsart University, Bangkok 10900, Thailand; preecha.pa@ku.th (P.P.); fscikpm@ku.ac.th (K.M.); 3 Department of Fishery Products, Faculty of Fisheries, Kasetsart University, Bangkok 10900, Thailand; kangsadan.b@ku.th; 4 Kasetsart University International College, Kasetsart University, Bangkok 10900, Thailand; amornthep.ki@ku.th; 5 Jiangsu Key Laboratory for Animal Genetic, Breeding and Molecular Design, Yangzhou University, Yangzhou 225009, China; yuzhang@yzu.edu.cn

**Keywords:** *Monascus purpureus*, comparative genomics, metabolism, pigment, protein function

## Abstract

*Monascus* sp. NP1 is a significant filamentous fungus with valuable properties for food industries. Initially isolated from the fermented rice product ang-kak, this strain is known for its ability to produce natural pigments. In this study, we therefore sequenced its genome together with the 26S rRNA D1/D2 domain and ITS fragment for identifying species of *Monascus* sp. NP1, and further conducted functional annotations of its overall genes related to metabolic capability and growth adaptation using comparative genomics. As a result, promisingly, the NP1 strain was identified as *Monascus purpureus* with the genome sequences, which was shown to be 23.54 Mb with a GC content of 49.01%. Genome annotation predicted 8031 protein-encoding genes. Comparative genomics between NP1 and 11 other related strains revealed 6024 core groups, 2204 accessory groups, and 5 strain-specific groups. Metabolic pathway analysis promisingly showed carbohydrate metabolism as the most enriched category, particularly central carbon metabolism involving key precursors, e.g., acetyl-CoA and pyruvate that support energy generation and the biosynthesis of pigments, fatty acids, and lipids. These findings highlighted the metabolic versatility and adaptive growth potential of *M. purpureus* NP1. This study provides key genetic insights into the cellular functions of *M. purpureus* NP1, laying the groundwork for exploring metabolic properties. It offers a comprehensive understanding for developing targeted applications of *M. purpureus* NP1 as an alternative fungal cell factory in food and nutrition.

## 1. Introduction

*Monascus* sp., a filamentous fungus, is widely used in food and agriculture, and also shows potential applications in pharmaceutical and health-related fields [1]. The widely recognized fermentation product, red yeast rice (ang-kak), is commonly used as a natural food colorant, meat and fish preservative, and a starter culture for brewing rice wine and vinegar [2,3]. *Monascus purpureus* is an important fungus in industrial biotechnology and is best known for its production of natural pigments [4]. In addition, it can produce a variety of other metabolites, such as exopolysaccharides and short-chain fatty acids (SCFAs) [5,6,7,8].

In our previous study, *Monascus* sp. strain NP1 was isolated from ang-kak and shown to produce pigments, indicating strong potential for sustainable biotechnological applications [9,10,11]. Given the growing interest in its bioactive properties, such as anti-oxidant, anti-microbial, anti-inflammatory, and anti-cancer activities [2,3], a comparative genomic analysis with other strains is necessary to identify strain-specific traits. To date, eleven genomes of *M. purpureus* are available in the NCBI database, including YY-1, the first reference genome widely used as a commercial strain [2], along with GB01, HQ1, YJX-8, CSU-M183, PF1702S, P7048X-2, RP2, KUPM5, M-32, and MP2022 strains [4,5,12,13]. Comparative analysis would clarify phylogenetic relationships, functional genes related to niche adaptation, and strain-level genomic diversity [12]. However, the genome of the *Monascus* sp. strain NP1 has not yet been sequenced, and its phylogenetic relationship, strain-specific function and metabolic potential remain uncharacterized.

Therefore, this study aimed to decipher the *Monascus* sp. strain NP1 genome and its unique functions in relation to metabolic capability and growth adaptation. Comparative genomic analysis of *Monascus* sp. strain NP1 with available *M. purpureus* genomes provides novel insights into the identification of species, common characteristics, and strain-specific traits. This study shows that *Monascus* sp. strain NP1 can be a potential fungal cell factory for functional foods and feeds, contributing to the future development of eco-friendly and commercially viable solutions.

## 2. Results

### 2.1. Identified Species of Monascus sp. NP1 and Its Genomic Characteristics

**Figure 1:**
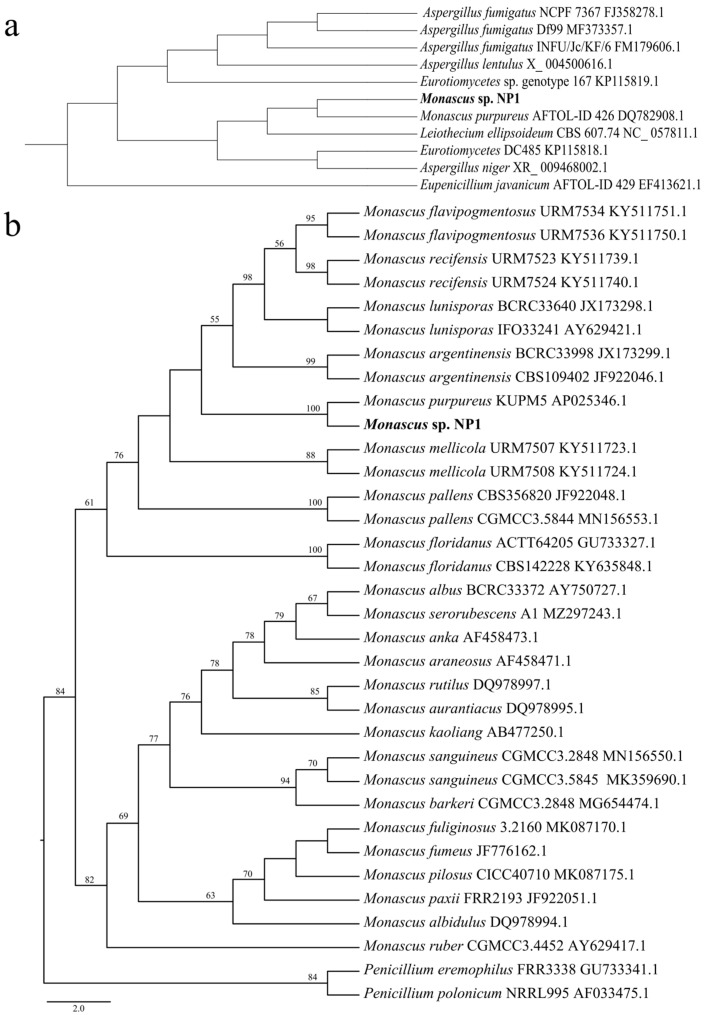
Sequence identification of *Monascus* sp. NP1. (**a**) Similarity-based analysis of 26S rRNA D1/D2 domains between NP1 and related fungal species using BLAST. (**b**) Phylogenetic analysis of ITS sequences of related *Monascus* species using the ML method. Bootstrap values ≥ 50% are illustrated in the Figure.

To identify species of *Monascus* sp. NP1, we analyzed the 26S rRNA D1/D2 domains using BLAST 2.17.0 (http://www.ncbi.nlm.nih.gov/BLAST/) (accessed on 1 July 2025), while ITS fragments were analyzed by constructing a phylogenetic tree based on reference sequences from 31 strains representing 23 *Monascus* species (Table S1). Regarding the 26S rRNA sequence data, the strain NP1 interestingly exhibited the highest sequence identity with *M. purpureus* strain AFTOL-ID 426 (97.70%) (Figure 1a, Table S2). For ITS analysis, this supports that NP1 was the closest related to *M. purpureus* strain KUPM5, which clustered into a single branch with a high bootstrap value of 100.00% using the maximum likelihood (ML) method (Figure 1b). Although these results did not converge on a single reference strain, which is generally a discrepancy in closely related taxa due to differences in resolution and evolutionary rate, both consistently support assigning *Monascus* sp. NP1 to *M. purpureus* at the species level.

**Figure 2:**
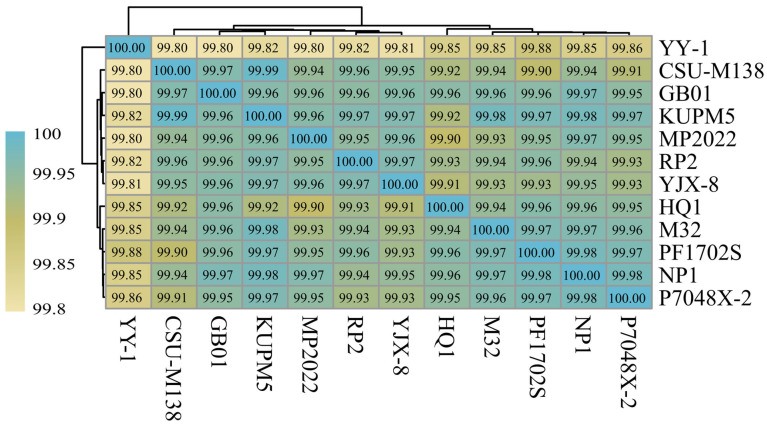
Hierarchical clustering heatmap of genome-wide ANI among *M. purpureus* strains. The color scale represents the ANI similarity values between genomes. Strain abbreviations correspond to the names of the analyzed *M. purpureus* isolates. Dendrograms indicate the clustering relationships based on ANI distances.

To further support the classification of NP1 as *M. purpureus* at the genomic level, Average Nucleotide Identity (ANI) analysis was also performed. In this study, ANI values among the strains exceeded 99.00%, indicating high genomic similarity. Specifically, ANI values between NP1 and the other 11 *M. purpureus* strains ranged from 99.85% to 99.98%, with the highest similarity (99.98%) observed for strains, e.g., P7048X-2, PF1702S, and KUPM5 (Figure 2). These results are consistent with the identification based on molecular markers, further supporting the classification of NP1 as *M. purpureus*.

To characterize the genome of *M. purpureus* NP1, genome assembly, annotation, and analysis were performed. As summarized in Table 1, the assembled genome has a size of 23.54 Mb with a GC content of 49.01% and contains 8031 protein-encoding genes. The assembly showed high completeness (98.40%) and good continuity, indicating a reliable genome for further analysis.

### 2.2. Comparative Genomic Features of M. purpureus NP1 with Those of Closely Related Strains

**Figure 3:**
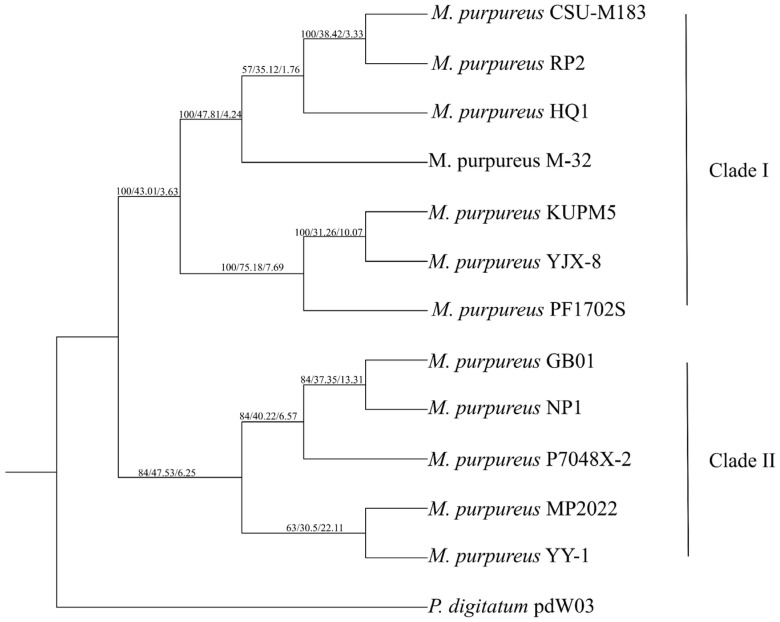
Illustration of a phylogenomic tree based on orthologous gene analysis. Note: This tree was constructed using single-copy orthologous gene (SCOG) analysis. Numbers at nodes represent bootstrap support (bootstrap), site concordance factor (sCF), and gene concordance factor (gCF) in ordering bootstrap/sCF/gCF, respectively.

Upon comparing the genomic features of *M. purpureus* NP1 with closely related strains, it was evident that the strains came from diverse sources, e.g., red rice (ang-kak), soil, feces, and a wine factory as shown in Table 2. Accordingly, the comparative genome sizes ranged from 23.22 to 24.53 Mb, for which the strain YJX-8 showed the largest genome (24.53 Mb) and the strain HQ1 showed the smallest genome (23.22 Mb). Based on the reported genome sizes, %GC content remains relatively similar across all strains, ranging from 49.0% to 49.5%. Moreover, a phylogenomic tree based on orthologous gene analysis also showed a high degree of genetic similarity of *M. purpureus* strains (Figure 3). Phylogenetic analysis divided these strains into different branches, i.e., NP1 clustering with GB01, P7048X-2, MP2022, and YY-1, belonging to Clade II.

### 2.3. Identified Core Genes and Strain-Specific Genes in M. purpureus NP1 Across the Other Strains

**Figure 4:**
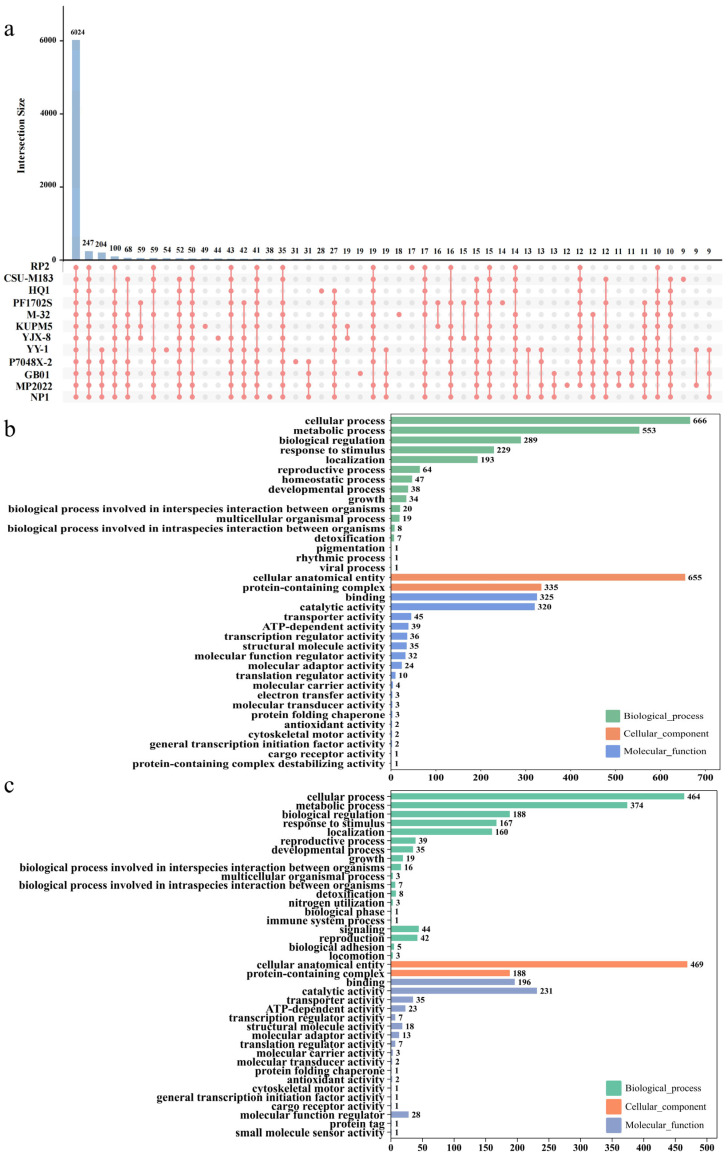
Assigned groups and strain-specific genes identified through orthologous clustering using OrthoFinder, with functional annotation, across all studied strains of *M. purpureus*. (**a**) UpSet plot showing the distribution of groups and strain-specific genes across all strains. Strain abbreviations correspond to the names of the analyzed *M. purpureus* isolates. The filled dots and connecting lines indicate the intersections among different strains, while the bar plot represents the number of assigned groups and strain-specific genes in each intersection. (**b**) GO annotation of core groups. (**c**) GO annotation of accessory groups.

To further investigate the core and strain-specific genes of *M. purpureus* NP1, all genomes of *M. purpureus* strains were performed by comparative orthologous gene analysis. As a result, a total of 91,835 protein-encoding genes from all genomes were analyzed using OrthoFinder v2.5.5. Of these, 91,507 genes (99.6%) were categorized into 8233 assigned groups (6024 core groups, 2204 accessory groups, and 5 strain-specific groups), while 328 genes (0.4%) remained unassigned in the Upset plot (Figure 4a). These unassigned genes may represent strain-specific sequences at the gene level, as they were not clustered into any groups.

Among the core groups of *M. purpureus* strains, 4047 core genes were categorized into three main GO categories in NP1, i.e., biological process (2170 genes), cellular component (990 genes), and molecular function (887 genes). GO annotation analysis highlighted the top four sub-GO terms including cellular process (666 genes), cellular anatomical entity (655 genes), metabolic process (553 genes), and protein-containing complex (335 genes) (Figure 4b, Table S3). To further investigate functional characteristics, GO enrichment analysis was performed. The core genome showed significant enrichment in molecular functions, i.e., sequence-specific DNA binding, sequence-specific double-stranded DNA binding, nucleic acid binding, and transition metal ion transmembrane transporter activity. Enriched cellular components included the transmembrane transporter complex, DNA replication preinitiation complex, nuclear pre-replicative complex, and pre-replicative complex. Biological processes significantly enriched included aspartate family amino acid metabolic and biosynthetic processes, lysine biosynthetic process via aminoadipic acid, and gene expression (Table S4).

In the accessory groups, a total of 1567 groups were identified in *M. purpureus* NP1, of which 2807 genes were analyzed by GO annotation, belonging to biological processes (1579 genes), cellular components (657 genes), and molecular functions (571 genes). The top four sub-GO terms include cellular anatomical entities (469 genes), cellular processes (464 genes), metabolic processes (374 genes), and catalytic activity (231 genes) (Figure 4c, Table S3). Subsequent GO enrichment analysis revealed significantly enriched molecular functions including thiolester hydrolase activity, kinase activator activity, protein kinase activator activity, and iron ion binding. Enriched cellular components included the endoplasmic reticulum sub-compartment, U5 snRNP, endoplasmic reticulum membrane, and organelle inner membrane, while enriched biological processes included the siderophore metabolic process, siderophore biosynthetic process, cardiolipin metabolic process, and cytochrome complex assembly (Table S5).

For strain-specific genes, 38 genes were identified in NP1, among which 20 genes were functionally annotated. These genes were involved in metabolism (11 genes), transmembrane transport (3 genes), protein metabolism, mitochondria and mitochondrial-related function (5 genes), and autophagy (1 gene) (Figure 4a; Table 3, Tables S6 and S7). Among these genes, four genes were further highlighted based on functional annotation, including *NP1-FUN_005870* encoding carbonic anhydrase (CtnG) (EC: 4.2.1.1), *NP1-FUN_001153* encoding satratoxin biosynthesis SC1 cluster protein 4-like (PF20684), *NP1-FUN_006508* encoding Zn (2)-C6 fungal-type DNA-binding domain superfamily (PF00172), and *NP1-FUN_003290* encoding NADH dehydrogenase (ubiquinone) 1β subcomplex subunit 9 (PF05347).

**Figure 5:**
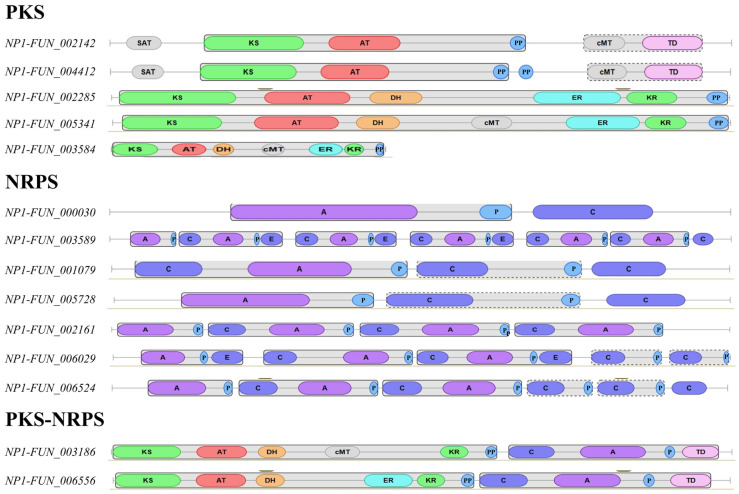
The protein domains of PKS, NRPS, and PKS-NRPS in *M. purpureus* NP1. Note: SAT, starter unit ACP transacylase; KS, ketosynthase; AT, acyltransferase; DH, dehydratase, cMT, carbon methyltransferase; ER, enoylreductase; KR, keto reductase; PP, phosphopantetheine acyl carrier protein group; TD, Terminal reductase domain; A, adenylation domain; P, peptidyl-carrier protein domain; C, condensation domain; and E, epimerization domain.

Considering that *NP1-FUN_001153* was annotated as a satratoxin biosynthesis SC1 cluster protein 4-like, further analysis was conducted to investigate whether a corresponding biosynthetic gene cluster is present in NP1. As shown in Table S8, a partial gene cluster (17 genes) was identified in NP1 based on BLAST analysis, exhibiting relatively low sequence similarity compared to the known satratoxin biosynthetic gene cluster (21 genes) from *Stachybotrys chartarum*. The antiSMASH fungi v6.1.1 analysis revealed multiple biosynthetic gene clusters in NP1, including PKS-encoding genes (5 genes), non-ribosomal peptide synthetase (NRPS)-encoding genes (7 genes), and PKS-NRPS hybrid genes (2 genes) (Figure 5). Taken together, these results suggest the presence of diverse secondary metabolite biosynthetic potential in NP1, but do not support the presence of a complete satratoxin biosynthetic pathway.

### 2.4. Identified Overall Metabolic Functions of M. purpureus NP1

**Figure 6:**
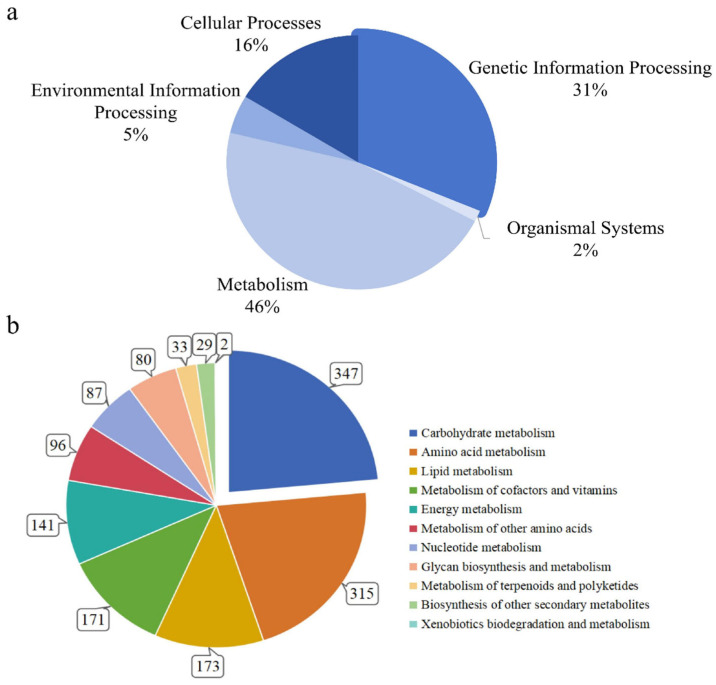
Illustration showing overall functional assignment of *M. purpureus* NP1. (**a**) Distribution of assigned protein functions into five main groups under KEGG database. (**b**) Distribution of metabolic genes in sub-metabolic functional categories.

The overall metabolic functions of 8031 protein-encoding genes were assigned using KEGG categories. Here, we classified 2414 genes (30.05% of the total) into five main groups, i.e., metabolism (46%), genetic information processing (31%), cellular processes (16%), environmental information processing (5%), and organismal systems (2%) (Figure 6a, Table S9). For metabolism, the highest numbers of genes were distributed into the top-three sub-metabolic categories, i.e., carbohydrate metabolism (347 genes), amino acid metabolism (315 genes), and lipid metabolism (173 genes) (Figure 6b). The remaining sub-categories were metabolism of cofactors and vitamins (171 genes), energy metabolism (141 genes), metabolism of other amino acids (96 genes), nucleotide metabolism (87 genes), glycan biosynthesis and metabolism (80 genes), metabolism of terpenoids and polyketides (33 genes), biosynthesis of other secondary metabolites (29 genes), and xenobiotics biodegradation and metabolism (2 genes). Carbohydrate metabolism included pyruvate metabolism (60 genes), glycolysis/gluconeogenesis (57 genes), starch and sucrose metabolism (53 genes), amino sugar and nucleotide sugar metabolism (49 genes), and glyoxylate and dicarboxylate metabolism (41 genes) (Figure S1a). For amino acid metabolism, notable contributions were observed in arginine and proline metabolism (64 genes), glycine, serine and threonine metabolism (61 genes), cysteine and methionine metabolism (54 genes), tryptophan metabolism (52 genes), and phenylalanine metabolism (50 genes) (Figure S1b). Lipid metabolism included glycerophospholipid metabolism (53 genes), glycerolipid metabolism (35 genes), fatty acid degradation (30 genes), steroid biosynthesis (27 genes), and ether lipid metabolism (16 genes) (Figure S1c).

To better understand the metabolic potential and evolutionary significance of *M. purpureus* NP1, we compared its metabolic genes with the closely related *Monascus* species and strains, and other filamentous fungi. Across *M. purpureus* strains, genes involved in primary metabolism (carbohydrate, amino acid, and lipid) were highly similar. In contrast, genes related to terpenoid/polyketide and other secondary metabolite pathways varied among strains, with NP1 showing slightly higher numbers (Table S10).

At the genus level, *M. purpureus* NP1, *M. ruber* BC20, and *M. pilosus* MS-1 showed similar gene distributions across major metabolic categories, especially in carbohydrate, amino acid, and lipid metabolism. Compared with other filamentous fungi (e.g., *Aspergillus* and *Penicillium*), they had higher gene numbers in several categories, particularly carbohydrate and amino acid metabolism and secondary metabolite biosynthesis (Table S11).

### 2.5. Explored Metabolic Capability and Growth Adaptation of M. purpureus NP1

**Figure 7:**
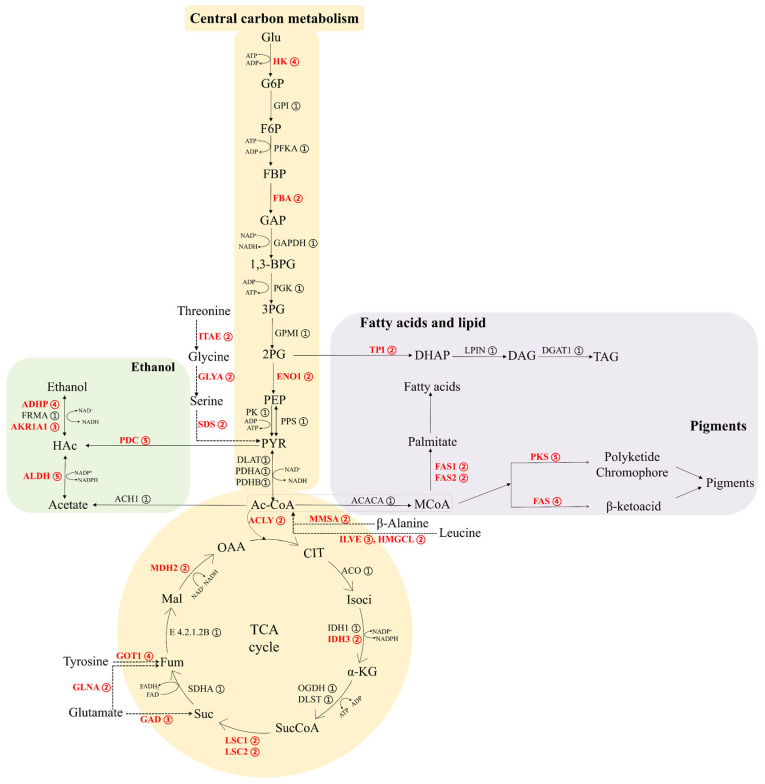
Schematic diagram illustrating the key metabolic capabilities of *M. purpureus* NP1, including central carbon metabolism, pigment biosynthesis, fatty acid and lipid biosynthesis, and ethanol production. Note: Colored boxes indicate different metabolic pathways: yellow for central carbon metabolism, purple for fatty acid and lipid biosynthesis, and green for ethanol production. Circled numbers represent gene copy numbers, and bold red letters indicate multi-copy genes. Dashed arrows denote lumped reactions. Full metabolite names are provided in Table S13.

To explore the metabolic capacity and growth adaptation of *M. purpureus* NP1, genes were categorized according to their copy number. Table 4 summarizes the enzymes encoded by multi-copy genes, while the complete dataset, including both single-copy and multi-copy genes, is presented in Table S12. Metabolic pathways were subsequently reconstructed to depict overall gene copy numbers and the distribution of enzymes (Figure 7). Regarding metabolic energy and supporting cell growth, central carbon metabolism, e.g., glycolysis and the TCA cycle, was the focus. In glycolysis, hexokinase (*HK*, 4 copies), fructose-bisphosphate aldolase (*FBA*, 2 copies), and enolase (*ENO1*, 2 copies) were identified. In the TCA cycle, isocitrate dehydrogenase (NAD+) (*IDH3*), malate dehydrogenase (*MDH2*), ATP citrate (pro-S)-lyase (*ACLY*), and alpha and beta subunits of succinyl-CoA synthetase (*LSC1* and *LSC2*) were each detected with two gene copies. Notably, multi-copy genes were also identified in amino acid metabolism, including aspartate aminotransferase in tyrosine metabolism (*GOT1*, 4 copies), glutamine synthetase in arginine biosynthesis (*GLNA*, 2 copies), glutamate decarboxylase in glutamate metabolism (*GAD*, 3 copies), L-threonine ammonia-lyase, glycine hydroxymethyltransferase, threonine aldolase in glycine, serine, and threonine metabolism (*SDS*, *GLYA*, and *ITAE*, 2 copies each, respectively), methylmalonate-semialdehyde dehydrogenase in β-alanine metabolism (*MMSA*, 2 copies), and branched-chain amino acid aminotransferase and hydroxymethylglutaryl-CoA lyase in leucine and isoleucine metabolism (*ILVE*, 3 copies; *HMGCL*, 2 copies, respectively).

In pigment biosynthesis, gene copy number analysis in *M. purpureus* NP1 identified five PKS and four fatty acid synthase (FAS) genes. It also supports fatty acids (*FAS1* and *FAS2*, 2 copies each) and lipid biosynthesis, including glycerone phosphate formation via triosephosphate isomerase (*TP1*, 2 copies). To further investigate the evolutionary relationships of these genes, phylogenetic analyses were performed using PKS and FAS sequences from NP1 and 11 additional *M. purpureus* strains. The resulting ML trees showed that PKS sequences from different strains clustered into well-supported clades rather than grouping by strain (Figure S2a). A similar pattern was observed for FAS genes, where *FAS1* and *FAS2* homologs from different strains grouped into distinct clades (Figure S2b), suggesting conserved evolutionary relationships across strains.

In ethanol biosynthesis, multiple gene copies were identified in key steps, including pyruvate decarboxylase (*PDC*, 5 copies), alcohol dehydrogenase (*ADHP*, 4 copies), NADP^+^-dependent alcohol dehydrogenase (*AKR1A1*, 3 copies), and aldehyde dehydrogenase (*ALDH*, 5 copies), indicating the presence of both direct and alternative pathways for ethanol production.

## 3. Discussion

The genus *Monascus* is widely used in red yeast rice fermentation and the brewing industry due to its ability to produce natural pigments and various bioactive compounds [5,7,8]. Species identification is essential because they exhibit distinct metabolic characteristics [12]. The ITS region and the 26S rRNA D1/D2 domains are commonly used molecular markers for fungal taxonomy [15,16]. In this study, phylogenetic analysis, based on ITS sequences and BLAST comparison of 26S rRNA sequences, both supported the classification of NP1 within *M. purpureus*. However, these molecular markers showed incongruence in the closest reference strains identified, inferring their resolution and evolution rate for closely related taxa [17]. ANI analysis was further performed to determine its taxonomic status at the genomic level. The ANI values between NP1 and other *M. purpureus* strains exceeded 99.85%, well above the species demarcation threshold (~95%) [18], indicating high genomic similarity. 

The NP1 genome exhibits typical features of *M. purpureus*, with a genome size of 23.54 Mb and a GC content of 49.01%, comparable to other reported strains (Table 2), indicating broadly genomic similarity across strains a conserved genomic structure and overall genomic stability across strains. Phylogenomic analysis showed NP1 within Clade II, clustering closely with GB01, P7048X-2, and MP2022. Notably, NP1 grouped with MP2022, a known ethanol-producing strain [5,10,11]. This phylogenetic relationship may suggest shared features related to fermentation-associated metabolism among Clade II strains [19]. However, this inference requires further functional and transcriptomic validation.

Comparative genomic analysis revealed a high proportion of core genes in *M. purpureus*, which indicates a conservation of essential cellular functions. These core genes were mainly involved in DNA binding and amino acid biosynthesis, highlighting their fundamental roles [20]. In addition, accessory genes were associated with catalytic activity, transport, and specialized metabolism, suggesting roles in environmental adaptation and metabolic flexibility [21]. This pattern is consistent with findings in *Aspergillus* species, where accessory genes contribute to lineage-specific adaptation and secondary metabolism [22]. Several strain-specific genes identified in NP1 may contribute to its distinctive metabolic features. For instance, the C*tnG* gene has been predicted to be involved in pathways related to malonyl-CoA, a key precursor for secondary metabolite biosynthesis [23], while Zn2-Cys6 transcription factor may be involved in the regulation of pigment biosynthesis [24]. In addition, a gene annotated as encoding a satratoxin biosynthesis SC1 cluster protein 4-like was identified in NP1. However, these functional assignments require further investigation for secondary biosynthetic genes and their roles contributing to NP1 metabolism. Given that biosynthetic gene clusters associated with secondary metabolites typically contain key enzymes, such as polyketide synthases and related components [25], this finding drove further investigation of PKS-related genes in NP1. Multiple PKS, NRPS, and PKS-NRPS hybrid genes were detected, suggesting a potential for secondary metabolite production. However, the specific functions of these gene clusters remain to be experimentally validated. Furthermore, genes related to mitochondrial electron transport, such as NADH dehydrogenase (ubiquinone) 1 beta subcomplex subunit 9, are associated with energy metabolism [26,27]. Given the importance of NAD^+^/NADH balance in ethanol fermentation, these genes may be associated with the potential for ethanol production in NP1 under certain conditions [28]. Overall, the analysis of core, accessory, and strain-specific genes highlights their roles in conserved cellular processes, environmental adaptation, and metabolic diversity, providing insights into the genetic basis of pigment and ethanol production in NP1. Focusing metabolic analysis provided additional insights into the metabolic features of *M. purpureus* NP1. It exhibited slightly higher gene numbers in terpenoid/polyketide metabolism and secondary metabolite biosynthesis, which may be associated with its ability to produce diverse metabolites. It also showed a metabolic gene profile similar to *M. pilosus* and *M. ruber*, both known for pigment production [29,30,31]. Overall, these results suggest that NP1 retains a conserved primary metabolism while exhibiting potential for diverse secondary metabolite production.

Analysis of gene copy numbers in *M. purpureus* NP1 provides insights into its metabolic potential and adaptability. Multiple gene copies in central carbon metabolism, including glycolysis and the TCA cycle, may contribute to the genetic capacity for the production of key intermediates such as pyruvate and acetyl-CoA and energy generation [32,33]. As a central metabolic node, acetyl-CoA links primary metabolism to the biosynthesis of secondary metabolites, ethanol, fatty acids, and lipids [34,35]. The presence of multiple genes involved in acetyl-CoA generation, such as *ACLY* and amino acid metabolism-related enzymes, may be associated with an increased genetic potential for acetyl-CoA production [36,37,38]. In pigment biosynthesis, acetyl-CoA is converted to malonyl-CoA for polyketide production [39,40]. NP1 contains multiple PKS and FAS genes that are conserved across *M. purpureus* strains, suggesting that these gene families are evolutionarily stable and maintained across strains rather than arising from lineage-specific expansion. Compared with other filamentous fungi, the moderate number of PKS genes in *M. purpureus* may reflect differences in secondary metabolite potential [41]. Acetyl-CoA is also involved in lipid metabolism. The presence of *FAS1*, *FAS2*, and *TPI* suggests the metabolic potential for lipid biosynthesis and energy storage [34,42]. Environmental factors such as glucose and oxygen availability may influence carbon allocation among competing pathways [43]. In addition, the strain-specific gene *CtnG*, encoding carbonic anhydrase, may contribute to metabolic regulation by influencing CO_2_ availability, which has been linked to acetyl-CoA levels and lipid accumulation [44]. Furthermore, ethanol biosynthesis represents another potential metabolic route for carbon utilization. The relatively high copy numbers of genes such as *PDC*, *ADHP*, and *ALDH* suggest a potential capacity for ethanol production [45,46,47]. Previous studies have shown that glucose concentration can influence ethanol production, with higher levels generally favoring ethanol accumulation [10,11,47]. In this context, the presence of multiple ethanol-related genes in NP1 may provide a genomic basis for ethanol production under specific conditions, although this requires further experimental validation. Overall, these results suggest that NP1 may possess a coordinated metabolic network centered on acetyl-CoA, enabling flexible carbon allocation among pigment, fatty acid, lipid, and ethanol biosynthesis pathways. This metabolic flexibility may contribute to its adaptability and potential industrial relevance. Future studies integrating transcriptomics, metabolomics, and metabolic flux analysis may further elucidate the regulatory mechanisms underlying these metabolic processes.

## 4. Materials and Methods

### 4.1. DNA Extraction Towards Genomic Sequencing of Monascus sp. NP1

*Monascus* sp. NP1 was maintained in the strain collection from the Faculty of Fisheries, Kasetsart University, Thailand. It was isolated from ang-kak (Chinese red rice) [9]. For cultivation, the strain was grown in 50 mL of glucose medium broth containing 0.025 g yeast extract, 0.5 g peptone, 1 g glucose, and 50 mL distilled water in a 250 mL flask. The inoculum was prepared using a spore suspension at a concentration of 1 mL (10^6^–10^7^ CFU/mL), following the method by Boonprab and Matsui [10,11]. The culture was incubated at 30 °C for 5 days with rotary shaking at 75 rpm. Genomic DNA was extracted using the QIAamp DNA Mini Kit (Qiagen, Hilden, Germany), and its quality was assessed using a NanoDrop One spectrophotometer (Thermo Scientific, Waltham, MA, USA) followed by gel electrophoresis. Whole-genome sequencing of *Monascus* sp. NP1 was performed by Novogene using an Illumina NovaSeq 6000 platform. The genomic sequence data of *Monascus* sp. NP1 was deposited in BioProject: PRJNA1165499, locus tag: ACFKG4, and accession number: JBISTQ000000000.

### 4.2. Identification of Species of Monascus sp. NP1

The 26S rRNA D1/D2 domain and ITS fragment were used to identify species of *Monascus* sp. strain NP1. The amplification of the 26S rRNA D1/D2 domain was conducted using primers LR0R (5′-ACCCGCTGAACTTAAGC-3′) and LR7 (5′-TACTACCACCAAGATCT-3′). Purification of the resulting PCR products was achieved with the QIAquick PCR Purification Kit (Qiagen, Hilden, Germany), followed by sequencing performed by Macrogen, Inc. (Seoul, Republic of Korea). Due to the limited availability of 26S rRNA reference sequences for *Monascus* species in public databases, the obtained DNA sequences were compared with known 26S rRNA gene sequences in GenBank using the BLAST, employing a threshold of ≥90% sequence identity and an E-value cutoff of 1 × 10^−5^. Additionally, the ITS fragment was extracted from the genome sequence of *Monascus* sp. NP1 using Get_Organelle v1.7.1 [48]. Given the broader availability and higher resolution of ITS sequences for species-level discrimination within the genus *Monascus*, ITS sequences were aligned with those of related 23 *Monascus* species (*M. purpureus*, *M. anka*, *M. ruber*, *M. fumeus*, *M. albidulus*, *M. paxii*, *M. pilosus*, *M. fuliginosus*, *M. kaoliang*, *M. serorubescens*, *M. rutilus*, *M. albus*, *M. araneosus*, *M. barkeri*, *M. sanguineus*, *M. aurantiacus*, *M. flavipogmentosus*, *M. lunisporas*, *M. recifensis*, *M. pallens*, *M. mellicola*, *M. argentinensis*, and *M. floridanus*) across 31 strains and 2 *Penicillium* species (*Penicillium polonicum* and *P. eremophilus*) as outgroups which were retrieved from the NCBI database (https://www.ncbi.nlm.nih.gov/) (accessed on 1 March 2025) using Geneious R11 [49] (Table S1). Species identification was further supported by phylogenetic analysis using the ML method. The ML phylogenetic tree was constructed using IQ-TREE v2.2.6 with automatic model selection, and branch support was assessed with 1000 bootstrap replicates [50]. Furthermore, ANI values among *M. purpureus* genomes were calculated using fastANI (v1.33) with the parameter-fragLen set to 500 bp [51]. Strains with an ANI value of ≥95% were used as the classification threshold to distinguish species. These ANI values were then utilized to construct a heat map using TBtools v2.012 [52].

### 4.3. Genome Assembly and Sequence Annotation

Genome assembly was initiated by assessing read quality using FastQC v0.12.1 [53]. Adapters and low-quality bases were removed with Trimmomatic version 0.39, applying the default parameters [54]. Reads with a minimum length of 36 bases were retained, and bases at the 3′ end with a quality score below Q20 were removed. De novo genome assembly was performed using SPAdes v4.0.0 [55]. Assembly quality and statistics, e.g., N50 and L50 values, longest scaffold were evaluated using QUAST v5.2.0 [56]. Genome completeness was evaluated using BUSCO v5.0.0 (fungi_odb10) [57]. Repetitive sequences were identified using RepeatModeler v2.0.1 for a de novo repeat library [58], followed by masking with RepeatMasker v4.1.5 [59]. For genome annotation, it was carried out using Funannotate v1.8.15 [60] for predicting protein-encoding genes. Furthermore, the genomes of eleven *M. purpureus* strains (KUPM5, YY-1, YJX-8, RP2, MP2022, CSU-M138, GB-01, M-32, P7048X-2, PF1702S, and HQ1) were obtained from the NCBI database (https://www.ncbi.nlm.nih.gov/) (accessed on 1 October 2024). Previous annotations employed different software and methodologies, which might result in variation in the number of predicted protein-encoding genes across studies [4,12]. To ensure consistency and enable reliable comparative genomic analysis, all genomes were enhanced using Funannotate v1.8.15, standardizing functional predictions across all studied strains.

As a form of protein sequences, comparative genomic analysis and phylogenetic analysis were explored. To achieve a phylogenomic tree, inference from all protein-encoding genes under OrthoFinder analysis was employed with default settings [61], using *Penicillium digitatum* pdW03 as an outgroup. For the evolutionary relationship, a phylogenomic tree was inferred from alignment of 4320 single-copy orthologous genes (SCOGs) using MAFFT v7.520 [62], with the removal of low-quality alignment regions and improvement of data quality using Gblocks (http://phylogeny.lirmm.fr/phylo_cgi/one_task.cgi?task_type=gblocks) (accessed on 1 March 2025), as well as the construction of a maximum-likelihood phylogenetic tree using the amino acid substitution model (VT + F + I + R8), with node support assessed through 1000 bootstrap replicates using IQ-TREE v2.2.6. Gene concordance factor (gCF) and site concordance factor (sCF) were calculated in IQ-TREE to assess gene-tree and site-level supports for each internal branch [63], respectively.

### 4.4. Identification of Core and Strain-Specific Genes Between Monascus sp. NP1 and the Other M. purpureus Strains

To identify core and strain-specific genes between *Monascus* sp. NP1 and the other eleven *M. purpureus* strains, OrthoFinder v2.5.5 was used to conduct an all-against-all sequence similarity search among predicted protein-encoding genes in a different analysis including only *M. purpureus* strains for orthogroup classification. This analysis was conducted independently from the phylogenomic analysis. In the OrthoFinder workflow, the Markov clustering algorithm was employed to generate orthogroups. Based on the OrthoFinder output, protein-encoding genes were then classified into the following assigned groups, i.e., core groups (present in all strains), accessory groups (present in multiple strains, but not all), strain-specific groups (orthogroups present in only one strain with multiple genes). The gene counts for each strain were then generated and analyzed using the UpSetR package v1.4.0 [64].

### 4.5. Dissection of Metabolic Functions of Monascus sp. NP1 and Its Unique Features

To dissect metabolic functions of *Monascus* sp. NP1, the functional annotation of protein-encoding genes was conducted with eggNOGmapper v6.0, which provides Gene Ontology (GO) terms and Kyoto Encyclopedia of Genes and Genomes (KEGG) pathway categories [65]. GO enrichment analysis was performed with a *p*-value threshold of <0.05 to identify significant functions. Protein-coding sequences were also analyzed with InterProScan release 105.0 (accessed on 1 October 2025) to predict protein domains and functional sites [66]. Secondary metabolite biosynthetic gene clusters (BGCs) were identified using antiSMASH fungi v6.1.1 [67] for PKS and NRPS detection, and BLAST analysis for the satratoxin gene cluster (SC1–SC21) based on protein sequences from the reference strain *Stachybotrys chartarum* IBT 7711 [25]. Metabolic pathway analysis was performed using the KEGG via BlastKOALA (www.kegg.jp/blastkoala) (accessed on 1 October 2025) release 115.0 (July 2025) [68,69]. To further explore unique features of *Monascus* sp. NP1, the list of strain-specific genes and the list of high gene copy numbers (≥2 genes) were analyzed for all possible protein functions and metabolic reactions, respectively. In addition, functional and pathway annotation was performed to assess the metabolic capacity of the NP1 strain in comparison with other *M. purpureus* strains, *Monascus* species, e.g., *M. ruber* (GCA_042920445) and *M. pilosus* (GCA_018806995), and other fungal species, e.g., *Aspergillus nidulans* (GCA_000011425), *A. niger* (GCA_000002855), *A. fumigatus* (GCA_000002655), *Penicillium brevicompactum* (GCA_028827555), and *P. chrysogenum* (GCA_028827035). Further comparative analysis focused on the metabolic category, with particular emphasis on differences in gene counts related to primary and secondary metabolic pathways among the species and strains explored. Furthermore, the putative PKS and FAS genes were identified from the genomes of NP1 and 11 other strains. To investigate the evolutionary relationship of these two gene families, phylogenetic analysis was performed using sequences from all 12 strains. Protein sequence alignment was performed using MAFFT v7.520 with default parameters. The ML phylogenetic tree was constructed using IQ-TREE v2.2.6 with automatic model selection, and 1000 bootstrap replicates were performed to assess branch support. To determine the root of the phylogenetic tree, appropriate outgroup sequences from relevant fungal species were included.

## 5. Conclusions

In conclusion, this study provides a genome analysis of *M. purpureus* NP1, revealing its genomic features and inferred metabolic potential based on genome annotation. Comparative genomics with related fungal species and strains identified genes potentially involved in pigment biosynthesis, as well as fatty acid and lipid biosynthesis, proposing the metabolic capacity of this strain. These functional assignments are notably based on genomic annotation data which requires further experimental validation. Future studies integrating multi-omics approaches would help elucidate gene regulatory mechanisms and refine our understanding of metabolic processes in NP1. These findings provide a basis for further exploration of *M. purpureus* NP1, including its potential applications in food, pharmaceuticals, and bioenergy, and identify candidate genomic targets that may inform future optimization efforts aimed at enhancing the production of its primary and secondary metabolites.

## Figures and Tables

**Table 1:** Genomic characteristics of *M. purpureus* NP1.

Genome Characteristics	Features
Total length (Mb)	23.54
GC content (%)	49.01
Scaffolds	294
N50 value	204,221
L50 value	36
Completeness of genome assemblies (BUSCO, %)	98.40
Percentage of repetitive elements (%)	4.48
Fold coverage (x)	117
Longest scaffold	710,344
Average length (bps)	79,800
Number of protein-encoding genes	8031

**Table 2:** Comparative genomic characteristics, sources, and production of *M. purpureus* strains. The strain in bold represents the strain analyzed in this study.

Strain	Accession	Sources	Country	Genome Size (Mb)	GC%	Productions	References
**NP1**	**This study**	**Red rice (ang-kak)**	**Thailand**	**23.54**	**49.01**	**Pigment, ethanol**	**[9,10,11]**
KUPM5	GCA_025999795	Thai fermented rice	Thailand	24.48	49.00	Pigment, citrinin, monacolin K	[14]
YY-1	GCA_003184285	Rice medium	China	24.15	49.00	Pigment, citrinin	[2]
YJX-8	GCA_011319195	Jiuqu	China	24.53	49.00	Pigment, short-chain fatty acid esters	[13]
RP2	GCA_023935125	Fermented food	China	24.40	49.00	Pigment	[12]
MP2022	GCA_031058495	Soil	China	24.40	49.50	Pigment, ethanol	[9]
CSU-M183	GCA_019320005	-	China	23.75	49.10	Pigment, citrinin, chitin	[12]
GB-01	GCA_004359145	-	Japan	24.30	49.00	Pigment	[4]
M-32	GCA_027497415	Red rice	China	23.63	49.00	Pigment	NCBI
P7048X-2	GCA_023624895	Human feces	China	23.30	49.00	Pigment, citrinin	[12]
PF1702S	GCA_023624875	Human feces	China	23.23	49.00	Pigment, citrinin	[12]
HQ1	GCA_006542485	Wine	China	23.22	49.00	Pigment, chitin	[12]

**Table 3:** List of strain-specific genes in *M. purpureus* NP1.

Gene Name	Protein Family/Protein Domain	Pfam ID/EC Number
*NP1-FUN_000900*	tRNA-specific adenosine deaminase 1	EC:3.5.4.34
*NP1-FUN_006751*	Major facilitator superfamily	PF07690
*NP1-FUN_003952*	Autophagy-related protein 5	PF20637
*NP1-FUN_007064*	MIOREX complex component 11	PF10306
*NP1-FUN_005870*	Carbonic anhydrase (CtnG)	EC:4.2.1.1
*NP1-FUN_004997*	Dolichol phosphate-mannose biosynthesis	PF07297
*NP1-FUN_005433*	Transient receptor potential calcium channel	-
*NP1-FUN_000759*	Xaa-Pro aminopeptidase P	PF01321
*NP1-FUN_004286*	Metallo-beta-lactamase domain-containing protein	PF07690
*NP1-FUN_001153*	Satratoxin biosynthesis SC1 cluster protein 4-like	PF20684
*NP1-FUN_003654*	MICOS complex subunit Mic12	PF17050
*NP1-FUN_001201*	Mitochondrial Tim protein assembly helper	-
*NP1-FUN_003290*	NADH dehydrogenase (ubiquinone) 1 beta subcomplex subunit 9	PF05347
*NP1-FUN_007973*	Pseudouridine-5′-phosphate glycosidase	PF04227
*NP1-FUN_001689*	Dynactin subunit 6	-
*NP1-FUN_004057*	Large ribosomal subunit protein mL53	PF10780
*NP1-FUN_004206*	E3 ubiquitin-protein ligase Zswim2	PF13639
*NP1-FUN_006508*	Zn (2)-C6 fungal-type DNA-binding domain superfamily	PF00172
*NP1-FUN_000287*	U4/U6.U5 small nuclear ribonucleoprotein 27kDa protein	PF08648
*NP1-FUN_005329*	Transcription factor IIIC, 90kDa subunit, N-terminal	PF12657

**Table 4:** Key metabolic pathways associated with high gene copy numbers in *M. purpureus* NP1.

Group	Pathway	Gene ID	EC Number	Enzyme Name	Gene Copy Number
Central carbon metabolism	Glycolysis	*NP1-FUN_000910*	2.7.1.1	Hexokinase (HK)	4
		*NP1-FUN_000443*			
		*NP1-FUN_000303*			
		*NP1-FUN_000130*			
		*NP1-FUN_001787*	4.1.2.13	Fructose-bisphosphate aldolase (FBA)	2
		*NP1-FUN_006964*			
		*NP1-FUN_000255*	4.2.1.11	Enolase (ENO1)	2
		*NP1-FUN_006878*			
	TCA cycle	*NP1-FUN_003303*	1.1.1.41	Isocitrate dehydrogenase (NAD^+^) (IDH3)	2
		*NP1-FUN_006569*			
		*NP1-FUN_003518*	6.2.1.4	Succinyl-CoA synthetase beta subunit (LSC2)	2
		*NP1-FUN_003847*			
		*NP1-FUN_003392*	6.2.1.5	Succinyl-CoA synthetase alpha subunit (LSC1)	2
		*NP1-FUN_003960*			
		*NP1-FUN_007858*	2.3.3.8	ATP citrate (pro-S)-lyase (ACLY)	2
		*NP1-FUN_007859*			
		*NP1-FUN_002477*	1.1.1.37	Malate dehydrogenase (MDH2)	2
		*NP1-FUN_002824*			
Amino acid metabolism	Tyrosine metabolism	*NP1-FUN_000463*	2.6.1.1	Aspartate aminotransferase (GOT1)	4
		*NP1-FUN_000811*			
		*NP1-FUN_004060*			
		*NP1-FUN_006289*			
	Arginine biosynthesis	*NP1-FUN_000350*	6.3.1.2	Glutamine synthetase (GLNA)	2
		*NP1-FUN_005489*			
	Glycine, serine and threonine metabolism	*NP1-FUN_001300*	4.3.1.19	L-threonine ammonia-lyase (SDS)	2
		*NP1-FUN_004791*			
		*NP1-FUN_000082*	2.1.2.1	Glycine hydroxymethyltransferase (GLYA)	2
		*NP1-FUN_005028*			
		*NP1-FUN_000540*	4.1.2.48	Threonine aldolase (ITAE)	2
		*NP1-FUN_001781*			
	β-Alanine metabolism	*NP1-FUN_004104*	1.2.1.18	Methylmalonate-semialdehyde dehydrogenase (MMSA)	2
		*NP1-FUN_007468*			
	Leucine and isoleucine degradation	*NP1-FUN_002062*	2.6.1.42	Branched-chain amino acid aminotransferase (ILVE)	3
		*NP1-FUN_006018*			
		*NP1-FUN_006781*			
		*NP1-FUN_001241*	4.1.3.4	Hydroxymethylglutaryl-CoA lyase (HMGCL)	2
		*NP1-FUN_004102*			
	Alanine, aspartate and glutamate metabolism	*NP1-FUN_004385*	4.1.1.15	Glutamate decarboxylase (GAD)	3
		*NP1-FUN_005143*			
		*NP1-FUN_007977*			
Pigment, fatty acid and lipid metabolism	Pigment biosynthesis	*NP1-FUN_002142*	-	Polyketide synthase (PKS)	5
		*NP1-FUN_004412*			
		*NP1-FUN_002285*			
		*NP1-FUN_005341*			
		*NP1-FUN_003584*			
	Fatty acid and lipid biosynthesis	*NP1-FUN_004423*	2.3.1.86	Fatty acid synthase subunit alpha (FAS1)	2
		*NP1-FUN_005826*			
		*NP1-FUN_004422*		Fatty acid synthase subunit beta (FAS2)	2
		*NP1-FUN_005827*			
		*NP1-FUN_007537*	5.3.1.1	Triosephosphate isomerase (TPI)	2
		*NP1-FUN_007686*			
Ethanol production	Ethanol biosynthesis	*NP1-FUN_002704*	4.1.1.1	Pyruvate decarboxylase (PDC)	5
		*NP1-FUN_005152*			
		*NP1-FUN_005303*			
		*NP1-FUN_005624*			
		*NP1-FUN_006143*			
		*NP1-FUN_002146*	1.2.1.3	Aldehyde dehydrogenase (NAD^+^) (ALDH)	5
		*NP1-FUN_003173*			
		*NP1-FUN_005774*			
		*NP1-FUN_006002*			
		*NP1-FUN_006070*			
		*NP1-FUN_003624*	1.1.1.2	Alcohol dehydrogenase (NADP^+^) (AKR1A1)	3
		*NP1-FUN_004622*			
		*NP1-FUN_006793*			
		*NP1-FUN_002976*	1.1.1.1	Alcohol dehydrogenase (ADHP)	4
		*NP1-FUN_003747*			
		*NP1-FUN_005302*			
		*NP1-FUN_007629*			

## Data Availability

Raw sequencing data, and genome assembly and annotation data, are available in the National Center for Biotechnology Information (NCBI) repository under BioProject: PRJNA1165499 (locus tag: ACFKG4); accession number: JBISTQ000000000. All sequence data are also available via OSF at https://osf.io/vtmsd (accessed on 1 January 2026).

## Supplementary Materials

The following supporting information can be downloaded at: https://www.mdpi.com/article/10.3390/ijms27083670/s1.
