# Supplementary material for: Unveiling Metabolic Capability and Growth Adaptation of Monascus purpureus NP1 Through Genomic Sequencing and Comparative Analysis
Source: Int J Mol Sci. 2026 Apr 20;27(8):3670. doi: 10.3390/ijms27083670 (PMC13116585; doi:10.3390/ijms27083670)
Supplement: Supplementary file 1 [file ijms-27-03670-s001.zip › Supplementary Figure_final.pdf]

Unveiling metabolic capability and growth adaptation of *Monascus purpureus* NP1 through genomic sequencing and comparative analysis

Haisu Hu [1,†], Preecha Patumcharoenpol [2,†], Kangsadan Boonprab [3], Amornthep Kingkaw [4], Yu Zhang [5], Kamonporn Masawang [2] and Wanwipa Vongsangnak [2,4*]

1 Interdisciplinary Graduate Programs in Bioscience, Faculty of Science, Kasetsart University, Bangkok 10900, Thailand

2 Department of Zoology, Faculty of Science, Kasetsart University, Bangkok 10900, Thailand

3 Department of Fishery Products, Faculty of Fisheries, Kasetsart University, Bangkok 10900, Thailand

4 Kasetsart University International College, Kasetsart University, Bangkok 10900, Thailand

5 Jiangsu Key Laboratory for Animal Genetic, Breeding and Molecular Design, Yangzhou University, Yangzhou 225009, China

* Correspondence: author: wanwipa.v@ku.ac.th

† These authors contributed equally to this work.

Supplementary information

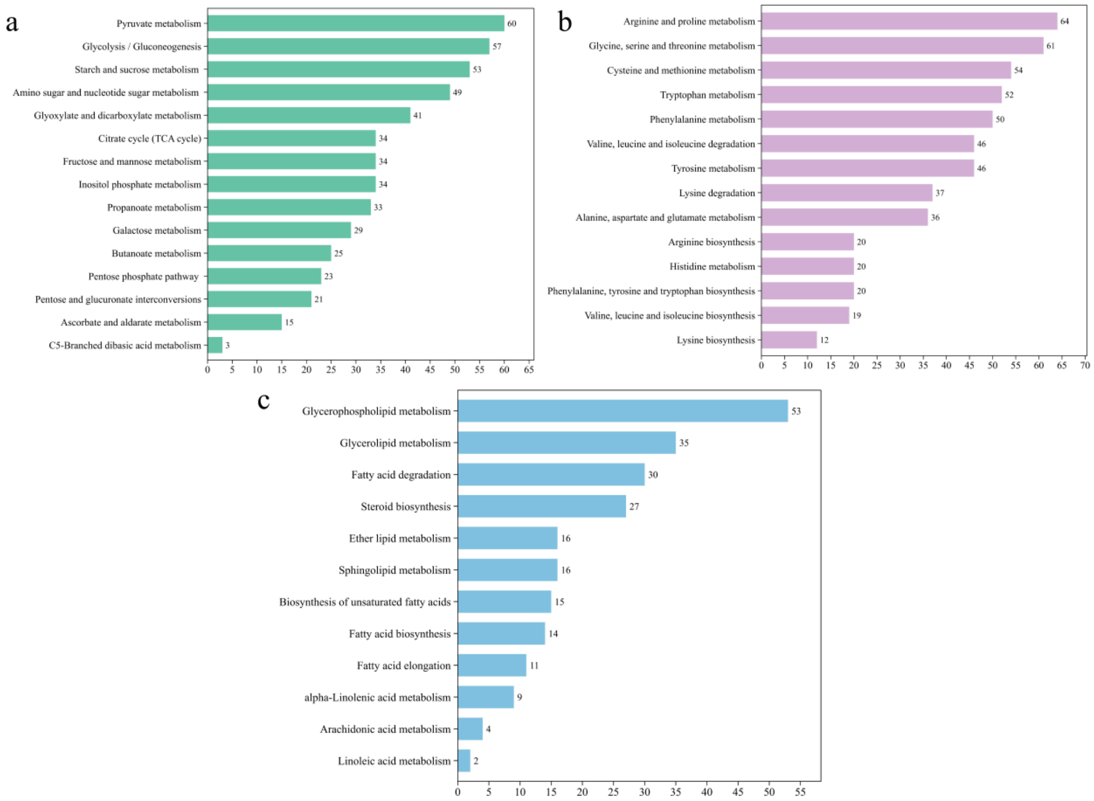

Figure S1. Distribution of metabolic gene numbers in *M. purpureus* NP1. (a) Carbohydrate metabolism, (b) Amino acid metabolism, and (c) Lipid metabolism.

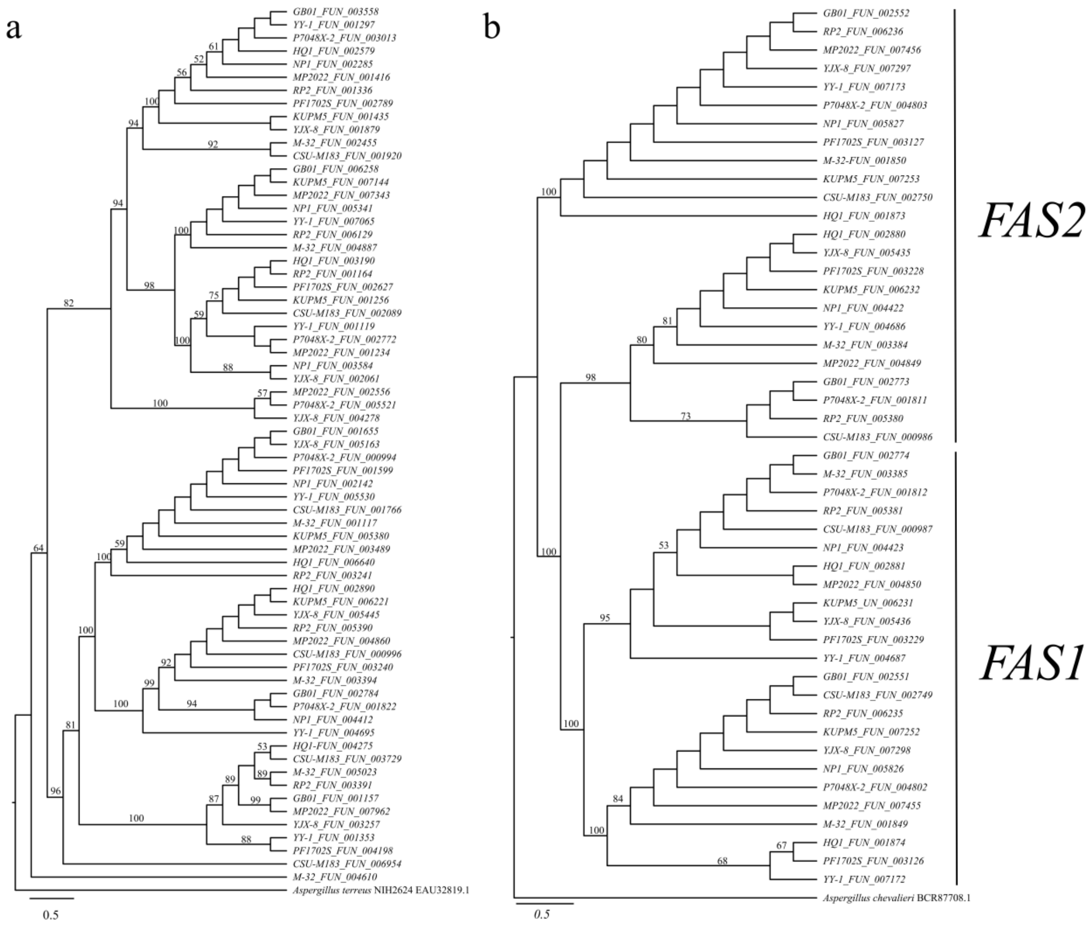

Figure S2. Phylogenetic analysis of PKS and FAS gene families in *Monascus purpureus*. (a) ML phylogenetic tree of PKS genes identified from *M. purpureus* NP1 and 11 additional strains. (b) ML phylogenetic tree of FAS genes (*FAS1* and *FAS2*) from *M. purpureus* NP1 and 11 additional strains. Bootstrap values ⩾50% are illustrated in the Figure.
